# Effects of Elevated Hydrostatic Pressure against Mesophilic Background Microflora and Habituated *Salmonella* Serovars in Orange Juice

**DOI:** 10.3390/microorganisms6010023

**Published:** 2018-03-09

**Authors:** Abimbola Allison, Edward Daniels, Shahid Chowdhury, Aliyar Fouladkhah

**Affiliations:** 1 Public Health Microbiology Laboratory, Tennessee State University, Nashville, TN 37209, USA; abimbolaallison20@gmail.com (A.A.); edwarddaniels258@gmail.com (E.D.); schowdh1@tnstate.edu (S.C.); 2 Cooperative Extension Program, Tennessee State University, Nashville, TN 37209, USA

**Keywords:** *Salmonella* serovars, mesophilic background microflora, orange juice, high pressure processing, elevated hydrostatic pressure

## Abstract

With recent improvements in the commercial feasibility of high pressure pasteurization units, the technology is gaining rapid acceptability across various sectors of food manufacturing, thus requiring extensive validation studies for effective adoption. Various times (1 min to 10 min) and intensity levels (0 MPa to 380 MPa) of elevated hydrostatic pressure were investigated for decontamination of mesophilic background microflora and inoculated *Salmonella* in orange juice. Results were analyzed by GLM procedure of SAS using Tukey- and Dunnett-adjusted ANOVA, additionally the K_max_ and D-values were calculated using best-fitted (maximum R^2^) model obtained by GInaFit software. At 380 MPa, for treatments of 1 min to 10 min, D-value of 1.35, and inactivation K_max_ of 3.34 were observed for *Salmonella* serovars. D-values were 5.90 and 14.68 for treatments of 241 MPa and 103 MPa, respectively. Up to 1.01 and >7.22 log CFU/mL reductions (*p* < 0.05) of habituated *Salmonella* serovars at planktonic stages were achieved using application of pressure at 380 MPa for 1 min and 10 min, respectively. Mesophilic background microflora counts were reduced (*p* < 0.05) by 1.68 to 5.29 log CFU/mL after treatment at 380 MPa for 1 min and 10 min, respectively. Treatments below two minutes were less efficacious (*p* ≥ 0.05) against the pathogen and background microflora, in vast majority of time and pressure combinations.

## 1. Introduction

Foodborne diseases can affect healthy individuals and more severely at-risk groups such as the very young, the elderly, pregnant women, and the immunocompromised [1]. It is estimated that around 30% of US population are currently considered as at-risk individuals for foodborne diseases [2]. Foodborne *Salmonella* infections are a major public health concern in the country and around the globe. From 1998 to 2016 more than 2500 foodborne outbreaks in the United States were associated with *Salmonella* serovars, leading to about 6900 cases of illnesses and >7900 hospitalization episodes [3]. 

Active surveillance data of the Centers for Disease Control and Prevention (CDC) indicates around 42,000 laboratory confirmed cases of non-typhoidal salmonellosis occur every year in the United States, with 94% of cases associated with contaminated food products, and leading to hospitalization in 27% of cases. Considering the unreported and under-diagnosed cases, CDC further estimates that non-typhoidal *Salmonella* serovars are responsible for 644,786 to 1,679,667 cases of foodborne illnesses resulting in 8545 to 37,490 annual episodes of hospitalization in the United States [4]. The bacterium is currently considered as the leading etiological agents of foodborne hospitalizations and deaths in the United States [4]. In addition to illness, hospitalization, and death episodes, many survivors of salmonellosis suffer long-term health complications such as reactive arthritis and post-infectious irritable bowel syndrome. Considering these health complications, captured in public health metrics such as Disability-Adjusted Life Years (DALYs), *Salmonella* serovars are the top-ranked pathogen of concern among major foodborne diseases [5]. 

The number of *Salmonella* outbreaks associated with contaminated fruits, vegetables, and juices have been increasing in recent years and have been a rising concern in public health since the early 1990s [6]. In response to these issues, the U.S. Food and Drug Administration (FDA) introduced regulations [7] which mandate that all 100% fruit/vegetable juices sold wholesale need to be produced under a Hazard Analysis and Critical Control Point (HACCP) plan. The regulation also requires processors to administer a treatment that results in at least a 5-log reduction of the most resistant microorganism of public health significance that are likely to occur in juices. Presently, *Salmonella* is commonly accepted as the pertinent pathogen that occurs in citrus juices [8]. In 1999, 2000, and 2005 there have been three multi-state outbreaks of *Salmonella* associated with orange juice in the United States [3]. Similar outbreaks in other countries such as India, Australia, Canada and South Africa had also been reported in the literature [8]. Various *Salmonella* serovars could survive up to 25 days in orange juice after manufacturing if product is untreated or not validated to inactivate the pathogen [9]. 

Emerging technologies have been investigated to replace or complement conventional interventions employed in food processing to prevent and eliminate foodborne diseases episodes and assure health of the public [10]. Although it had been target of research in academia for several decades [11], application of high pressure processing in food manufacturing had been gaining increasing momentum in recent years due to advances in engineering of commercially available units [10]. Currently, more than 100 different food products around the world are brought to market using this emerging technology [12], and in the United States the market size of pressure treated products are estimated to surpass $9B annually [13]. The technology is emerging response of the private industry for manufacturing of products with clean label, extended shelf-life, and fresh-like qualities some of the major demands of consumers in 21st century [10]. The National Advisory Committee on Microbiological Criteria for Foods (NACMCF) has therefore recommended that pasteurization be redefined, and high pressure processing has been recommended for the supplementary non-thermal pasteurization [14].

Considering the public health significance of Salmonellosis, and repeated involvement of juices in sporadic and outbreak foodborne disease episodes, the objective of this investigation is to conduct a laboratory challenge study for inactivation of *Salmonella* serovars and mesophilic background microflora in orange juice exposed to various time and intensity levels of elevated hydrostatic pressure for validating a safe, feasible, and efficacious decontamination hurdle in manufacturing.

## 2. Materials and Methods

### 2.1. Bacterial Inoculum Preparation

A five-strain mixture of habituated *Salmonella* serovars (ATCC^®^ numbers 13076, 8387, 6962, 9270, 14028) was used for inoculation of sterilized orange juice. Strains were existing in the Public Health Microbiology Laboratory of Tennessee State University and were originally acquired from American Type Culture Collection company (Manassas, VA, USA). The above-mentioned selected strains belong to *enterica* species of Salmonellae and represent sub-species of Enteritidis, Montevideo, Newport, Anatum, and Typhimurium, respectively. These were selected based on previous screening studies conducted in acidified foods [15]. The five selected sub-species also belong to the top 10 dominant *Salmonella* serovars of public health importance based on a 1998–2014 epidemiological study of the United States Department of Agriculture [16]. It is noteworthy that *Salmonella* is a highly diverse species with over 2500 serotypes and variability inevitably may exist in various strains’ susceptibility to intrinsic and extrinsic factors of food and food manufacturing. Current mixture of strains are selected based on laboratory challenge studies of foodborne isolates of public health significance conducted previously by current investigators [15] and are in harmony with the above-mentioned epidemiological evidence, thus could lead to generalizable discussions associated with *Salmonella* serovars of food and public health importance. 

For storage of each strain prior to conduct of the experiments, a loopful from frozen −80 °C glycerol/pathogen stock was aseptically transferred into 10 mL Tryptic Soy Broth (Difco, Becton Dickinson, Franklin Lakes, NJ, USA) with 0.6% yeast extract (Tryptic Soy Broth + yeast extract e.g., TSB + YE ), then incubated 20–24 h at 37 °C. A 100 μL of the above-mentioned overnight suspension was then streak plated onto the surface of Tryptic Soy Agar (Difco, Becton Dickinson, Franklin Lakes, NJ, USA) with 0.6% yeast extract (TSA + YE), and incubated at 37 °C for 24 h. The plates were then stored no more than a month at 4 °C prior to activation for the experiments. Five days prior to experiments, each strain was activated by culturing a single colony from the above-mentioned plates stored at 4 °C into 10 mL of sterile TSB + YE, followed by an incubation at 37 °C for 24 h. A 100 μL aliquot was then sub-cultured into another 10 mL TSB + YE and incubated again at 37 °C for 24 h. For each strain separately, cells from the sub-cultured overnight suspension (2000 μL aliquot) were harvested using centrifugal force at 6000 RPM (3548 g, for 88 mm rotor) for 15 min (Model 5424, Eppendorf North America, Hauppauge, NY, USA; Rotor FA-45-24-11). After discarding the supernatant, in order to further remove sloughed cell components, excreted secondary metabolites, and growth media, the cells were re-suspended in 2 mL Phosphate Buffered Saline (VWR International, Radnor, PA, USA) and re-centrifuged using the above-mentioned time and intensity. After removal of supernatant, to improve the external validity of the challenge study, each strain was then individually habituated in sterile orange juice for 72 h at 4 °C to allow acclimatization of the pathogen to low temperature and intrinsic factors of the product [17] prior to experiment. This was achieved by 10-fold dilution of the above-mentioned purified overnight culture in sterilized orange juice for target cell density of 8.5 log CFU/mL prior to habituation, and for yielding a target microbial load of 7.5 log CFU/mL after the 72-h habituation. On the day of experiments, habituated cells were vortexed (Model Vortex-2 Genie, Scientific Industries, Bohemia, NY, USA) for 60 s, then composited into a five-strain habituated mixture of the pathogen. The mixture was then 10-fold serially diluted and used for inoculation of the sterilized orange juice. Sterility of the orange juice prior to the inoculation was confirmed by plating 1 mL aliquot of the product onto surface of TSA + YE, incubated at 37 °C for 44–48 h. In separate experiments involving mesophilic background microflora, a product without any thermal or non-thermal treatment was used, purchased from a local supermarket. Use of TSB + YE for activation and sub-culturing of the bacterium were based on preliminary trials comparing multiplication and pH of the selected five strains in TSB supplemented with and without up to 0.6% yeast extract and 0.1% sodium pyruvate (data not shown). This medium had also been previously utilized to minimize acid stress during preparation of bacterial inoculum [11]. 

### 2.2. High Pressure Processing Treatment

**Figure 1:**
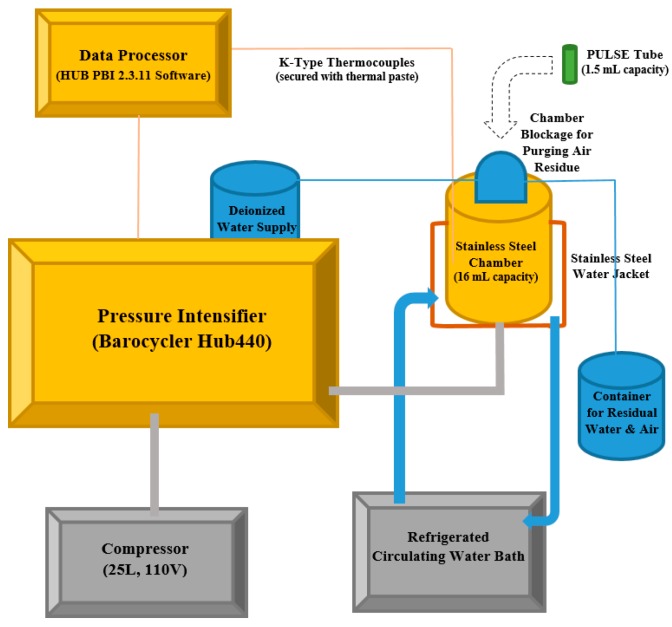
Schematic of high pressure processing unit utilized in current study at Public Health Microbiology laboratory, Tennessee State University. Hydrostatic pressure (Barocycler Hub440, Pressure Bioscience Inc., South Easton, MA, USA) applied in a 16 mL chamber surrounded by a stainless steel water jacket connected to a circulating water bath (Model refrigerated 1160s, VWR International, Radnor, PA, USA) for precise control of temperature. Temperature monitored by two k-type thermocouples (Omega Engineering Inc., Norwalk, CT, USA) inserted inside the wall of the chamber secured with thermal paste (Model 5 AS5-3.5G, Arctic Silver, Visalia, CA, USA) and connected to the unit’s software (HUB PBI 2.3.11 Software, Pressure BioScience Inc., South Easton, MA, USA). The treatments were conducted in no disk PULSE tubes (Pressure BioScience Inc., South Easton, MA, USA) containing 1.5 mL of habituated inoculum and the food vehicle.

Hydrostatic pressure (Barocycler Hub440, Pressure Bioscience Inc., South Easton, MA, USA) of 35 MPa to 380 MPa (15,000 to 55,000 pounds per square inch [PSI]) was applied at 0 (control) to 10 time intervals for decontamination of the inoculated pathogen. This study utilized moderate levels of hydrostatic pressure—higher levels of hydrostatic pressure could lead to higher cost associated with increased maintenance and decreased life of pressure vessels in private industry [18]. The treatment intensity levels were selected based on preliminary trials (data not shown) at moderate levels of importance to stakeholders of the technology and presented in both English and metric systems. The above-mentioned high pressure processing unit has a chamber size of 16 mL with the chamber surrounded by a stainless steel water jacket connected to a circulating water bath (Model refrigerated 1160s, VWR International, Radnor, PA, USA) for precise control of the temperature. To monitor the temperature, two k-type thermocouples (Omega Engineering Inc., Norwalk, CT, USA), were inserted inside the wall of the chamber, and then secured with thermal paste (Model 5 AS5-3.5G, Arctic Silver, Visalia, CA, USA) and connected to the unit’s software (HUB PBI 2.3.11 Software, Pressure BioScience Inc., South Easton, MA, USA). The temperature was recorded every three seconds by the software (data not shown) to assure conduct of experiments at controlled temperature of 25 °C. The pressure transmission fluid used in this study was distilled water (total dissolved solids < 30 PPM), chamber was purged prior to each treatment for removal of residual air in chamber’s headspace. The reported treatment time values exclude the time for pressure increase (come up time of 3 s) and the release time (come down time of 1 s), monitored using the Barocycler mode of HUB PBI 2.3.11 software (Pressure BioScience Inc., South Easton, MA, USA). These values, along with the above-mentioned temperature recordings were monitored and automatically recorded every 3 s using the HUB PBI 2.3.11 Software (data not shown). The treatments were conducted in no disk PULSE tubes (Pressure BioScience Inc., South Easton, MA, USA) containing 1.5 mL of habituated inoculum and the food vehicle. The PULSE tubes were then subjected to pressure treatments at 0, 5000 PSI (35 MPa), 15,000 PSI (103 MPa), 35,000 PSI (241 MPa) and 55,000 PSI (380 MPa), with a holding time of 1, 2, 4, 8, and 10 min in addition to non-treated controls (Time 0). Temperature was precisely controlled and monitored during the treatments at 25 °C. Schematic presentation of the unit is provided in Figure 1. 

### 2.3. Microbiological and pH Analyses

After treatments and prior to microbiological analyses, each sample was neutralized using D/E neutralizing broth (Difco, Becton Dickinson, Franklin Lakes, NJ, USA). Pressurized and control cell suspensions in PULSE tubes were then10-fold serially diluted in Maximum Recovery Diluent (Difco, Becton Dickinson, Franklin Lakes, NJ, USA), and spread plated onto TSA ± YE to enhance the recovery of injured cells during the inoculation study of sterile product and inactivation experiments of mesophilic background microflora. For enumeration of total aerobic bacteria, after incubation at 37 °C for 48 h, plated samples were manually counted and converted to log values for further analyses. The pH of all enumerated samples were measured after treatment using a digital pH meter calibrated at pH levels of 4, 7 and 10 prior to analyses (Mettler Toledo AG, Grelfensee, Switzerland).

### 2.4. Experimental Design and Statistical Analyses

The sample size of at least 5 samples per time/pressure was selected based on power analysis using *Proc Power* of SAS_9.2_ software (SAS Inst., Cary, NC, USA.), using existing preliminary data in Public Health Microbiology Laboratory of Tennessee State University, at statistical power of 80%, type one error level of 5%, and for detecting mean difference of 0.15 log CFU/mL as statistically significant. To achieve at least five sample size per analyses, each experiment was conducted in two biologically independent repetitions as two blocking factors in a complete randomized block design. Each block consisted of three replications per sample, and each analysis was conducted in two microbiological repetitions. Thus, overall each reported value is mean of 12 repetitions. Detection limit of this study was 0.35 log CFU/ml and experiments associated with inoculated pathogen and mesophilic background microflora were conducted separately, thus analyzed and reported independently. Initial data management and log transformation of the data was conducted using Microsoft Excel. A Generalized Linear Model, using Proc GLM of SAS was utilized for ANOVA analysis of the results. Further mean separation was conducted using Tukey-and Dunnett-adjusted ANOVA for pair-wise comparisons of treated samples, and comparisons of each treated sample with control, respectively. All analyses were conducted at type 1 error level of 5% (α = 0.05).

In order to calculate the inactivation rates associated with pressure treatments at 25 °C and to calculate linear and non-linear inactivation indices, GlnaFiT version 1.7 software (Katholieke Universiteit, Leuven, Belgium) was used. The software, as described by [19], reports inactivation rates of K_max_ expressed in the unit of 1/minute based on best-fitted (maximum R^2^) non-linear model. To obtain the index, log CFU/ml counts before exposure (control) and after 1, 2, 4, 8, and 10 min exposures at controlled temperature were analyzed using the software. Similarly, for D-value calculations, the time required for one log reduction (unit minute) were calculated using best-fitted linear model. Small values of K_max_ indicate longer treatment time required for cycles of log reduction of a bacterium while smaller D-values indicate less time required for one log reduction [15].

## 3. Results and Discussion

### 3.1. Sensitivity and Inactivation of Salmonella Serovars

**Figure 2:**
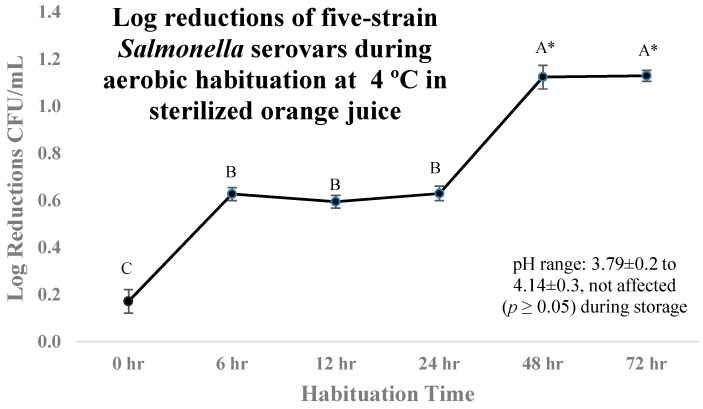
Number(s) of log reductions of five-strain mixture of *Salmonella* serovars (ATCC^®^ numbers 13076, 8387, 6962, 9270, 14028) inoculated in sterilized orange juice. Zero hour time shows the log reduction immediately after addition of the pathogen mixture to sterilized orange juices. Values followed by difference uppercase letters are statistically different (*p* < 0.05), resulted from pair-wise comparisons of all six time intervals (Tukey-adjusted ANOVA). Values followed by * are statistically different (*p* < 0.05) than control (8.68 ± 0.1 log CFU/mL), derived from comparisons of each time interval with the control (Dunnett-adjusted ANOVA).

The pH of the inoculated samples were ranging from 3.75 ± 0.1 to 4.06 ± 0.1 and were similar (*p* ≥ 0.05) for pressure treated samples. The pH of the untreated and non-inoculated orange juice were 3.86 ± 0.1 and was not different (*p* ≥ 0.05) than the treated samples. Habituation of samples were resulted in 1.1 log CFU/mL reductions (*p* < 0.05) of the bacterium (Figure 2) after 72 h of aerobic habituation at 4 °C. Habituation up to 24 h did not affected the counts of the inoculated bacterium (Figure 2). As further delineated in the *High pressure processing treatment* section, the temperature of the experiment were precisely controlled and monitored at 25 °C, thus the log reductions of the pathogen could be solely attributed to sensitivity to elevated hydrostatic pressure. Similarly, the inactivation indices were calculated based on reductions obtained at 25 °C and for a five-strain habituated mixture of *Salmonella* serovars, all belonging to *enterica* species and subspecies of Enteritidis, Montevideo, Newport, Anatum, and Typhimurium. 

**Figure 3:**
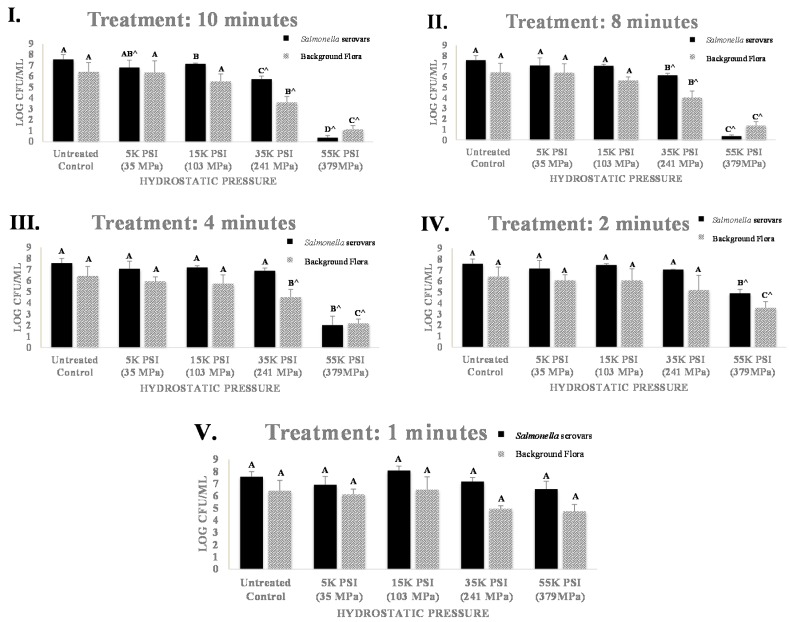
Effects of elevated hydrostatic pressure against five-strain habituated mixture of *Salmonella* serovars (ATCC^®^ numbers 13076, 8387, 6962, 9270, 14028) in sterilized orange juice and mesophilic background flora in non-sterile orange juice, treated (Barocycler Hub440, Pressure BioScience Inc., South Easton, MA, USA) at 25 °C For *Salmonella* serovars and mesophilic background flora separately. Columns of each time interval followed by different uppercase letters are representing log CFU/mL values that are statistically (*p* < 0.05) different (Tukey-adjusted ANOVA). Uppercase letters followed by ^ sign are statistically (*p* < 0.05) different than the control (Dunnett-adjusted ANOVA). (**I**). Treatment for 10 min; (**II**). Treatment for 8 min; (**III**). Treatment for 4 min; (**IV**). Treatment for 2 min. (**V**); Treatment for 1 min; Black solid bars represent counts (mean ± SD) of *Salmonella* serovars, patterned bars represent counts associated with mesophilic background flora. The pH of samples were ranging from 3.75 ± 0.1 to 4.06 ± 0.1 and were similar (*p* ≥ 0.05) among the various treated samples and control.

The target inoculation load of the pathogen were 7.5 log CFU/mL and yielded the pathogen counts of 7.58 ± 0.1 log CFU/mL for untreated samples after habituation. When exposed to very low levels of 35 MPa and 103 MPa (5000 and 15,000 PSI, respectively) of hydrostatic pressure, *Salmonella* serovars showed no appreciable sensitivity, particularly for treatments with durations of less than 4 min (Sections III, IV, and V of Figure 3).

For treatments at 10 min at the above-mentioned pressures, reductions (*p* < 0.05) of up to 0.75 log CFU/mL were observed, that is equal to approximately 82% reduction of the inoculated pathogen (Section I of Figure 3). Predictably, at higher levels of elevated hydrostatic pressure, these reductions were more biologically appreciable. For example, at 241 MPa (35,000 PSI), 0.53, 0.69, 1.44, and 1.88 log CFU/mL reductions were observed (*p* < 0.05) for treatments of 2, 4, 8, and 10 min, respectively (Sections I, II, III, and IV of Figure 3).

These reductions were appreciably higher at 380 MPa (55,000 PSI) were the pathogen was reduced (*p* < 0.05) to less than detection limit for 8- and 10-min treatments (Sections I and II of Figure 3), that is equal to >7 log CFU/mL reductions. Remaining treatments at 380 MPa were also efficacious (*p* < 0.05) for reduction of the pathogen, showing 1.01, 2.76, and 5.56 log reductions for 1-, 2-, and 4-min treatments, respectively (Sections III, IV, and V of Figure 3). These results are in harmony with existing literature were it had been demonstrated that treatments at 300 MPa and 400 MPa could result in about 5 log and >6 CFU/mL reduction in orange juice, respectively [20]. Similarly, past studies have showed more than 6 log CFU/mL reduction of *Salmonella* in orange juice by various treatments of up to 10 min at 300 MPa to 500 MPa at 30 °C [20]. It is noteworthy that exposure of foodborne bacterium to acidic environments such as orange juice, had been previously reported to induce acid tolerance response that could lead to cross-protective effects and increased resistance to further hurdles such as heat [21,22] and enhancements in biofilm formation capability of the pathogen [23]. Current study investigated effects of hydrostatic pressure at controlled temperature against bacterial mixture that were exposed to acidic environment for 72 h prior to treatment (Figure 2). Sensitivity of wild-type and acid-stressed phenotypes of foodborne pathogens to various combination of heat and elevated hydrostatic pressure is currently a gap in existing literature and an important component for robust risk assessments of Salmonellosis in acidic and acidified foods to be treated for safety and shelf stability by high pressure pasteurization. 

Inactivation indices such as D-value and K_max_ provide critical information for efficacy of a treatment for reducing a specific pathogen at specific intrinsic and extrinsic conditions [17]. This enables the practitioners to easily utilize the literature for validating an existing process, or researchers to compare inactivation efficacy of various thermal and non-thermal interventions. The D-value refers to decimal reductions and is the time required at given sets of conditions to achieve 90% reduction of the exposed microorganism [11,24,25]. As delineated earlier, D-values in this study were obtained using a linear model, and corresponds to log reductions associated with a five-strain habituated mixture of *Salmonella* serovars at 25 °C. The inactivation K_max_ reported in the current study is obtained from the best fitted (maximum R^2^) non-linear model obtained by GInaFiT software. Unlike the D-values that are reported in unit of minute, the inactivation K_max_ values have unit of 1/min, thus larger K_max_ values correspond to lower inactivation efficacy [19]. While some studies suggest indices obtained from non-linear models such as the first order inactivation constant K_max_ provide more accurate summary of an inactivation treatments relative to an index derived from a linear model [19,26], D-value continue to be a common reporting value due to its presence in food microbiology literature in last several decades [11,25].

**Figure 4:**
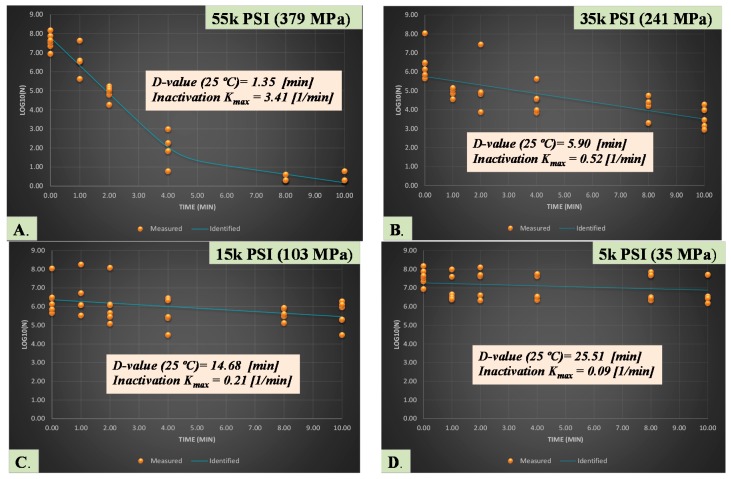
Inactivation rates for five-strain mixture (ATCC^®^ numbers 13076, 8387, 6962, 9270, 14028) of habituated *Salmonella* serovars exposed to elevated hydrostatic pressure (Barocycler Hub 440, Pressure BioScience Inc., South Easton, MA, USA) in sterilized orange juice. K_max_ values are selected from best fitted model (goodness-of-fit indicator of R^2^ values, α = 0.05) using GInaFiT software. K_max_ values are expression of number of log cycles of reduction in 1/min unit, thus larger values indicate less time required for microbial cell reductions in each tested level of hydrostatic pressure. D-values provided are calculated based best fitted linear model, indicating time required for one log (90%) of microbial cell reductions of the habituated microbial mixture. (**A**). Treatment at 55K PSI (380 MPa) with R^2^ = 0.97; (**B**). Treatment at 35K PSI (241 MPa) with R^2^ = 0.85; (**C**). Treatment at 15K PSI (103 MPa) with R^2^ = 0.74; (**D**). Treatment at 5K PSI (35 MPa) with R^2^ = 0.77.

Under the condition of this experiment, D-value for a treatment at 380 MPa (55,000K PSI) was 1.35 min indicating the treatment could lead to 90% reduction of the pathogen in orange juice at 25 °C every 1.35 min (Figure 4A). The corresponding first order inactivation constant (K_max_) obtained from non-linear model was 3.41 1/min. D-values and the inactivation K_max_ obtained from treatments at 241 MPa (35,000 PSI) were 5.90 min and 0.52 1/min, respectively, and were 14.68 min and 0.21 1/min for treatments at 103 MPa (15,000 PSI), respectively (Figure 4B,C). The later set of inactivation indices indicate that assuming the reduction following a linear pattern, a treatment for 14.68 min is required for 90% reduction of the pathogen at 103 MPa. With assumption of non-linearity of the reductions, only 0.21 (1/min) log cycle of reduction could be achieved using elevated pressure at this pressure level every minute of application at 25 °C. 

Previously, it has been demonstrated that high pressure processing at 600 MPa for 60 s at 20 °C could reduce inoculated *Salmonella* by at least 7 log CFU/mL [27]. Our study similarly shows 2.68, 5.56, and >7.23 log CFU/mL reductions of the pathogen after 2-, 4-, and 8-min treatments at 380 MPa (Sections II, III, and IV of Figure 3). Similar reductions, particularly those surpassing the 5-log regulatory requirement for HACCP validation [7], could be particularly favorable for manufacturers since higher pressure treatments had been associated to higher maintenance cost, lower vessel shelf-life, and higher initial capital investment [18]. Validated milder treatments with longer time duration could be feasible for small manufacturers who manufacture their products at smaller volumes, and those manufacturers that produce products with additional hurdles such as mild heat, and refrigeration requirement after production. Preservation of nutrients in a product could be additional benefit of treatments at lower pressure intensity, relative to pressure treatments at higher levels as well as thermal treatments. As an example, it had been demonstrated that pressure treatment (500 MPa, 35 °C, 5 min) retain up to 65% more vitamin C in orange juice during shelf-life compared to a conventional (80 °C, 30 s) heat treatment [28]. These losses of vitamin C and other bioactive compounds due to processing could potentially be further extenuated by using lower level of pressure that are validated to meet the safety and regulatory requirements of manufacturing. Literature discussing nutrient and bioactive loss as affected by various hydrostatic pressure levels appear to be a knowledge gap in hurdle validation studies associated with elevated pressure. Some studies indicate that mild pressures of 50 MPa to 350 MPa could potentially increase bioavailability of vitamin A in orange juice from 20–43% while not affecting the total radical scavenging capacity of the product [29]. Other studies similarly discuss pressures from 200 MPa to 300 MPa do not negatively affect the total phenolic compounds and total radical scavenging capacity of pressure-treated orange juice relative to untreated samples [30].

### 3.2. Sensitivity and Inactivation of Mesophilic Background Microflora

Prior to the treatment, the mesophilic background microflora of the orange juice was enumerated at 6.43 ± 0.9. Higher degree of dispersion in microbiological data in the current study, as evidence by the large standard deviation of the background flora counts relative to the pathogen count, are consistent to previous literature and could be attributed to diverse species in naturally contaminated products [31]. Mild pressure treatments of 35 MPa and 103 MPa (5000 PSI to 15,000 PSI) did not (*p* ≥ 0.05) reduced the mesophilic background microflora even when tested for 10 min (Figure 3). Pressure of 103 MPa is approximately >1000 times higher than the atmospheric pressure [32]. Similar treatments reduced up to 82% of the inoculated pathogen (Section I of Figure 3), showing less sensitivity of mesophilic background microflora, relative to inoculated pathogen. This could be associated with presence of bacterial spores that are ubiquitous in manufacturing and natural environments and are generally considered to be more resistant to elevated hydrostatic pressure treatments [33]. Predictably, higher levels of hydrostatic pressure resulted in more biologically significant reductions. Treatments at 241 MPa (35,000 PSI) were reduced the mesophilic background microflora by 2.80, 2.39, and 1.91 log CFU/mL when tested for 10, 8, and 4 min, respectively (Sections I, II and III of Figure 3). At 380 MPa (55,000 PSI), the reductions were ranging from 1.68 log CFU/mL at 1 min of treatment (Sections V of Figure 3) to 5.29 CFU/mL after a 10-min treatment (Sections V of Figure 3). These inactivation rates are equivalent to 97.9 to >99.9% reductions of the population of mesophilic background microflora. These findings are in harmony with existing literature were an approximately 2.5 log CFU/mL reductions of endogenous background microflora of orange juice were reported after 2 min of treatment at 350 MPa [34]. Our study observed a 2.84 log reductions (*p* < 0.05) for a 2-min treatment at 380 MPa (Section IV of Figure 3). It is noteworthy that current study only enumerates mesophilic background flora of the product after aerobic incubation. Spore forming microorganisms such as *Bacillus coagulans* and *Bacillus megaterium* and various species of *Alicyclobacillus* could survive a heat or high pressure treatment at low levels and limit the shelf-life of the product [35]. Thus, similar to a heat pasteurized product, a validation plan for a low-cost and efficacious high pressure processing of juices would require additional hurdles such as refrigeration or could have limited shelf-life compared to commercially sterilized products.

## 4. Conclusions

Under the conditions of this study, up to 1.01 and >7.22 log CFU/mL reductions (*p* < 0.05) of habituated *Salmonella* serovars at planktonic stage were achieved using application of pressure at 380 MPa for 1 to 10 min, respectively. Mesophilic background microflora counts were similarly reduced (*p* < 0.05) by 1.68 to 5.29 log CFU/mL after treatment at 380 MPa for 1 min and 10 min, respectively. Treatments at 35 MPa to 240 MPa were less efficacious (*p* ≥ 0.05), particularly when tested at time intervals below four minutes. Overall, mesophilic background microflora were less sensitive than the inoculated pathogen at vast majority of tested time and pressure intervals. This could be attributed to higher diversity of the organism presented in mesophilic background microflora relative to the five-strain habituated pathogen cocktail as well as potential presence of microbial spores that are ubiquitous in nature and resistant to pressure treatments. At 380 MPa, for treatments of 1 to 10 min, D-value of 1.35, and inactivation K_max_ of 3.34 were observed for *Salmonella* serovars treated at 25 °C. D-values were 5.90 and 14.68 for treatments of 241 MPa and 103 MPa, respectively. Inactivation indices obtained based on linear and non-linear models in this study show different reduction trends. For example, at 380 MPa, 1.35 min was required for 90% reduction of the pathogen when a linear model was consulted while 3.41 cycles of log reductions were achievable every minute based on interpretation of the non-linear model. This indicates that classical linear inactivation values, such as D-value, are not necessarily an accurate tool in predictive microbiology particularly when the inactivation curve of a pathogen-product combination possesses non-linear properties. Thus, commercial adoption, and interpretation of inactivation indices should be performed cautiously after statistically examining the properties of the product-pathogen inactivation curve.

Results of this study indicate that even without incorporation of thermal procedures, mild pressures could assure in excess of 5 log reductions of the *Salmonella* serovars, assisting a manufacturers meeting the safety and regulatory requirements of interstate and international commerce. Application of mild hydrostatic pressure could be of particular interest for reducing costs associated with processing maintenance, increasing pressure vessels shelf-life, and reducing initial capital investment. This could be a feasible and adoptable intervention particularly for products that have additional microbiological hurdle during process and/or supply chain such as acidic and acidified products and those requiring refrigeration after manufacturing. Existing literature also suggests pressures below 400 MPa typically leave no detectable quality and nutritional changes in many products, assisting a manufacturer achieving fresh-like qualities and clean label.

## References

[1] Fouladkhah A. (2017). The Need for Evidence-Based Outreach in the Current Food Safety Regulatory Landscape. Commentary section. J. Ext..

[2] Bajpai V.K., Baek K.H., Kang S.C. (2012). Control of *Salmonella* in foods by using essential oils: A review. Food Res. Int..

[3] Centers for Disease Control and Prevention (CDC) Foodborne Outbreak Online Database (FOOD Tool). https://wwwn.cdc.gov/foodborneoutbreaks/.

[4] Scallan E., Hoekstra R.M., Angulo F.J., Tauxe R.V., Widdowson M.A., Roy S.L., Jones J.L., Griffin P.M. (2011). Foodborne illness acquired in the United States—Major pathogens. Emerg. Infect. Dis..

[5] Scallan E., Hoekstra R.M., Mahon B.E., Jones T.F., Griffin P.M. (2015). An assessment of the human health impact of seven leading foodborne pathogens in the United States using disability adjusted life years. Epidemiol. Infect..

[6] Sivapalasingam S., Friedman C.R., Cohen L., Tauxe R.V. (2004). Fresh produce: A growing cause of outbreaks of foodborne illness in the United States, 1973 through 1997. J. Food Prot..

[7] Food and Drug Administration (FDA) 21 Code of Federal Regulations 120; CFR—Code of Federal Regulations Title 21. https://www.accessdata.fda.gov/scripts/cdrh/cfdocs/cfcfr/CFRSearch.cfm?CFRPart=120.

[8] Danyluk M.D., Goodrich-Schneider R.M., Schneider K.R., Harris L.J., Worobo R.W. Outbreaks of Foodborne Disease Associated with Fruit and Vegetable Juices, 1922–2010. http://ucfoodsafety.ucdavis.edu/files/223883.pdf.

[9] Foley D.M., Pickett K., Varon J., Lee J., Mln D.B., Caporaso R., Prakash A. (2002). Pasteurization of fresh orange juice using gamma irradiation: Microbiological, flavor, and sensory analyses. J. Food Sci..

[10] Rastogi N.K., Nguyen L.T., Jiang B., Balasubramaniam V.M. (2010). Improvement in texture of pressure-assisted thermally processed carrots by combined pretreatment using response surface methodology. Food Bioprocess. Technol..

[11] Balasubramaniam V.M., Barbosa-Cánovas G.V., Lelieveld H.L. (2016). High Pressure Processing of Food: Principles, Technology and Applications.

[12] Pinto C., Moreira S.A., Fidalgo L.G., Santos M.D., Delgadillo I., Saraiva J.A. (2016). Shelf-life extension of watermelon juice preserved by hyperbaric storage at room temperature compared to refrigeration. LWT-Food Sci. Technol..

[13] Pressure BioScience Inc. (PBI) High Pressure Processing. https://d1io3yog0oux5.cloudfront.net/_57b4ee00ca1d2b0fef872a3c158b2b76/pressurebiosciences/db/399/3062/pdf/Investor+Presentation+Oct+2017.pdf.

[14] Wang C.Y., Huang H.W., Hsu C.P., Yang B.B. (2016). Recent advances in food processing using high hydrostatic pressure technology. Crit. Rev. Food Sci. Nutr..

[15] Fouladkhah A., Geornaras I., Yang H., Sofos J.N. (2013). Lactic acid resistance of Shiga toxin-producing *Escherichia coli* and multidrug-resistant and susceptible *Salmonella* Typhimurium and Salmonella Newport in meat homogenate. Food Microbiol..

[16] United States Department of Agriculture-Food Safety Inspection Service (USDA-FSIS) (2014). Serotypes Profile of Salmonella Isolates from Meat and Poultry Products January 1998 through December 2014. https://www.fsis.usda.gov/wps/wcm/connect/3866026a-582d-4f0e-a8ce-851b39c7390f/Salmonella-Serotype-Annual-2014.pdf?MOD=AJPERES.

[17] Fouladkhah A., Geornaras I., Sofos J.N. (2012). Effects of Reheating against *Listeria monocytogenes* Inoculated on Cooked Chicken Breast Meat Stored Aerobically at 7 degree C. Food Prot. Trends.

[18] Manu D., Mendoca A., Daraba A., Dickson J., Sebranek J., Shaw A., Wang F., White S. (2017). Antimicrobial efficacy of cinnamaldehyde against *Escherichia coli* O157: H7 and *Salmonella enterica* in carrot juice and mixed berry juice held at 4 C and 12 C. Foodborne Pathog. Dis..

[19] Geeraerd A.H., Valdramidis V.P., Van Impe J.F. (2005). GInaFiT, A freeware tool to assess non-log-linear microbial survivor curves. Int. J. Food Microbiol..

[20] Velázquez-Estrada R.M., Hernández-Herrero M.M., López-Pedemonte T.J., Briñez-Zambrano W.J., Guamis-López B., Roig-Sagués A.X. (2011). Inactivation of *Listeria monocytogenes* and *Salmonella enterica* serovar Senftenberg 775W inoculated into fruit juice by means of ultra-high pressure homogenisation. Food Control.

[21] Koutsoumanis K.P., Sofos J.N. (2004). Comparative acid stress response of *Listeria monocytogenes*, *Escherichia coli* O157:H7 and Salmonella Typhimurium after habituation at different pH conditions. Lett. Appl. Microbiol..

[22] Mazzotta A.S. (2001). Thermal inactivation of stationary-phase and acid-adapted *Escherichia coli* O157:H7, Salmonella, and *Listeria monocytogenes* in fruit juices. J. Food Prot..

[23] Chorianopoulos N., Giaouris E., Grigoraki I., Skandamis P., Nychas G.J. (2011). Effect of acid tolerance response (ATR) on attachment of *Listeria monocytogenes* Scott A to stainless steel under extended exposure to acid or/and salt stress and resistance of sessile cells to subsequent strong acid challenge. Int. J. Food Microbiol..

[24] Xu H., Lee H.Y., Ahn J. (2009). High pressure inactivation kinetics of *Salmonella enterica* and *Listeria monocytogenes* in milk, orange juice, and tomato juice. Food Sci. Biotechnol..

[25] Jay J.M., Loessner M.J., Golden D.A. (2006). Modern Food Microbiology.

[26] Buchanan R.L., Golden M.H., Whiting R.C. (1993). Differentiation of the effects of pH and lactic or acetic acid concentration on the kinetics of *Listeria monocytogenes* inactivation. J. Food Prot..

[27] Bull M.K., Zerdin K., Howe E., Goicoechea D., Paramanandhan P., Stockman R., Sellahewa J., Szabo E.A., Johnson R.L., Stewart C.M. (2004). The effect of high pressure processing on the microbial, physical and chemical properties of Valencia and Navel orange juice. Innov. Food Sci. Emerg. Technol..

[28] Polydera A.C., Stoforos N.G., Taoukis P.S. (2003). Comparative shelf life study and vitamin C loss kinetics in pasteurised and high pressure processed reconstituted orange juice. J. Food Eng..

[29] De Ancos B., Sgroppo S., Plaza L., Cano M.P. (2002). Possible nutritional and health-related value promotion in orange juice preserved by high-pressure treatment. J. Sci. Food Agric..

[30] Velázquez-Estrada R.M., Hernández-Herrero M.M., Rüfer C.E., Guamis-López B., Roig-Sagués A.X. (2013). Influence of ultra-high pressure homogenization processing on bioactive compounds and antioxidant activity of orange juice. Innov. Food Sci. Emerg. Technol..

[31] Fouladkhah A., Geornaras I., Nychas G.J., Sofos J.N. (2013). Antilisterial Properties of Marinades during Refrigerated Storage and Microwave Oven Reheating against Post-Cooking Inoculated Chicken Breast Meat. J. Food Sci..

[32] Allison A., Chowdhury S., Fouladkhah A. Biofilm formation and decontamination of wild-type and pressure-stressed Cronobacter sakazakii and Salmonella serovars. Health and Medical Sciences Section. Proceedings of the 127th Meeting of Tennessee Academy of Science, University of Tennessee at Martin.

[33] Daryaei H., Balasubramaniam V.M. (2013). Kinetics of *Bacillus coagulans* spore inactivation in tomato juice by combined pressure–heat treatment. Food Control.

[34] Parish M.E. (1998). High pressure inactivation of *Saccharomyces cerevisiae*, endogenous microflora and pectinmethylesterase in orange juice. J. Food Saf..

[35] Yue T., Zhang J., Yuan Y. (2014). Spoilage by Alicyclobacillus bacteria in juice and beverage products: Chemical, physical, and combined control methods. Compr. Rev. Food Sci. Food Saf..

